# Immunological status and virological suppression among HIV-infected adults on highly active antiretroviral therapy

**DOI:** 10.1186/s12199-020-00881-6

**Published:** 2020-08-24

**Authors:** Mulugeta Melku, Gizachew Abebe, Amanuel Teketel, Fikir Asrie, Aregawi Yalew, Belete Biadgo, Eyuel Kassa, Debasu Damtie, Degefaye Zelalem Anlay

**Affiliations:** 1 Department of Hematology and Immunohematology, School of Biomedical and Laboratory Sciences, College of Medicine and Health Sciences, University of Gondar, Gondar, Ethiopia; 2 School of Biomedical and Laboratory Sciences, College of Medicine and Health Sciences, University of Gondar, Gondar, Ethiopia; 3 Department of Clinical Chemistry, School of Biomedical and Laboratory Sciences, College of Medicine and Health Sciences, University of Gondar, Gondar, Ethiopia; 4 University of Gondar comprehensive specialize referral Hospital, College of Medicine and Health Sciences, University of Gondar, Gondar, Ethiopia; 5 Department of Immunology and Molecular Biology, School of Biomedical and Laboratory Sciences, College of Medicine and Health Sciences, University of Gondar, Gondar, Ethiopia; 6 Food Animal Health Research Program, CFAES, Ohio Agricultural Research and Development Center, Department of Veterinary Preventive Medicine, The Ohio State University, Wooster, OH 44691 USA; 7 Global One Health Initiative, Eastern African Regional Office, The Ohio State University, Addis Ababa, Ethiopia; 8 Department of Community Health Nursing, School of Nursing, College of Medicine and Health Sciences, University of Gondar, Gondar, Ethiopia

**Keywords:** Adults, HAART, HIV/AIDS, Immunological status, Virological suppression

## Abstract

**Background:**

World Health Organization (WHO) recommends that viral load ([VL) is a primary tool that clinicians and researchers have used to monitor patients on antiretroviral therapy (ART), an antiviral drug against retroviruses. Whereas, CD4 cell counts can only be used to monitor clinical response to ART in the absence of VL testing service. Therefore, this study is aimed to assess the level of immunological status and virological suppression, and identify associated factors among human immunodeficiency virus ([HIV)-infected adults who were taking antiretroviral drugs of combination regimen know as highly active antiretroviral therapy (HAART).

**Methods:**

A hospital-based cross-sectional study was conducted at the University of Gondar comprehensive specialized referral hospital from February to April 2018. A total of 323 adult participants on HAART were selected using a systematic random sampling technique and enrolled into the study. Blood samples for viral load determination and CD4 cell count were collected. Binary logistic regression analysis was used to determine factors associated with immunologic status and virological suppression in HIV patients on HAART. Odds ratio with 95% CI was used to measure the strength of association.

**Results:**

Virological suppression (VL level < 1000 copies/ml) was found in 82% (95% CI 77.7, 86.1) of study participants, and it has been associated with CD4 cell count between 350 and 499 cells/mm^3^ (adjusted odds ratio (AOR) = 2.56; 95% CI 1.14, 5.75) and > 499 cells/mm^3^ (AOR = 7.71; 95% CI 3.48, 17.09) at VL testing and current age > 45 years old (AOR = 5.99; 95% CI 2.12, 16.91). Similarly, favorable immunological status (≥ 400 cells/mm^3^ for male and ≥ 466 cells/mm^3^ for female) was observed in 52.9% (95% CI 47.4, 58.8) of the study participants. Baseline CD4 cell count of > 200 cells/mm^3^, age at enrollment of 26 through 40 years old, and urban residence were significantly associated with favorable immunological status.

**Conclusion:**

Though the majority of HIV-infected adults who were on HAART had shown viral suppression, the rate of suppression was sub-optimal according to the UNAIDS 90-90-90 target to help end the AIDS pandemic by 2020. Nonetheless, the rate of immunological recovery in the study cohort was low. Hence, early initiation of HAART should be strengthened to achieve good virological suppression and immunological recovery.

## Introduction

The human immunodeficiency virus (HIV) pandemic continues to be a major public health threat in the world. Globally, more than 37.9 million people were living with HIV by the end of 2018. An estimated 70% of infections and deaths occur in the sub-Saharan region [1]. As a standard of care for HIV-infected individuals, the use of HAART reduces HIV-related morbidity and mortality [1, 2]. This treatment transforms HIV from rapidly fatal infection to a manageable chronic disease [3, 4]. The rapid scale-up of ART for treatment of HIV infection in resource-limited countries has been successfully saving the life of millions of people receiving HAART in low- and middle-income countries [5–7].

Ethiopia has made substantial progress in terms of HIV prevention and control activities [8]. It has been implementing a number of programs, including universal access to ART, to reduce the burden of HIV/AIDS-related complications [9]. It has also adopted the WHO’s 2016 consolidated guidelines on the use of antiretroviral drugs for treating and preventing HIV infection which include treating all people living with HIV/AIDS (PLWHA) (“Test and Treat” approach). The expansion in healthcare service delivery in general and HIV-related clinical services in particular had resulted a significant decline in the annual new HIV infection and HIV/AIDS-related deaths in the country. As per the Ethiopian Federal Ministry of Health report, there were 426,472 PLWHA on ART by the year 2016 [10].

The rapid scale-up of ART in resource-limited setting could be accompanied by an increased risk of HIV drug resistance which in turn could compromise the performance of the national ART roll-out program [11, 12]. Coupled with the rapid scale-up of ART services in sub-Saharan Africa, the need for appropriate treatment response monitoring approaches had become indispensable [13–15]. However, it becomes evident that monitoring of treatment response remains the key challenge for program managers and policymakers in Ethiopia due to sub-optimal access to HIV VL testing for routine follow-up of treatment [16–18].

In clinical practice, plasma HIV ribonucleic acid (RNA) level and CD4 cell count are used to monitor HIV treatment response. The relationship between plasma VL and CD4 cell count in HIV disease is complex. CD4 cell count is a commonly used marker of HIV disease progression and the key for starting prophylaxis and monitoring ART in the absence of HIV VL testing service. Sustained response to HAART and suppression of HIV VL were associated with an increase in CD4 cell counts [19]. The immunological and virological response to ART can be measured by the ability to suppress viral replication, time to suppression, the durability of suppression, and level of immune reconstitution achieved with suppression. These immunological and virological responses vary at individual and population levels [20].

WHO treatment guideline recommends the use of VL testing by clinicians and researchers as a primary tool to monitor treatment response in HIV-infected individuals on HAART. Whereas CD4 cell counts can only be used to monitor clinical response to HAART where VL testing is not available [21]. Besides, it is recommended that VL measurements among people receiving HAART should be performed at 6 months after initiating ART, then at 12 months, and every year thereafter [22]. Furthermore, one of the goals of the Joint United Nations Programme on HIV/AIDS (UNAIDS), “90-90–90 initiative”, is achieving virological suppression in 90% of all people receiving ART by the year 2020 [23]. Identifying factors that affect the virological suppression and designing appropriate strategies to mitigate the problem would have a paramount importance in terms of achieving the stated goal. Different studies indicated that factors such as poor adherence, low baseline CD4 cell count, low BMI, rural residence, advanced disease stage, and duration of treatment negatively affect the immunologic status and virological suppression and hinder the achievement of the targeted treatment goal [24–26].

In Ethiopia, immunologic and clinical parameters have been used to monitor HIV patients on ART. Recently, the government has implemented a VL testing in multiple testing centers across the regions; however, the viral suppression rate and immunological status are not well studied and even there is a need for locally generated data-driven evidence to inform policymakers and guide clinicians. Therefore, the aim of this study was to determine the immunologic and virological status and associated factors among adult PLWHA who were on HAART at the University of Gondar comprehensive specialized referral hospital.

## Methods

### Study design, setting, and period

An institutional-based cross-sectional study was conducted at the University of Gondar comprehensive specialized referral hospital ART clinic from February to April 2018. Gondar is located 750 km northwest of Addis Ababa, a capital city of Ethiopia. The University of Gondar comprehensive specialized referral hospital is a teaching hospital which serves for more than 5 million people. The hospital provides ART service since 2005. More than 7500 adults and 700 pediatric patients were enrolled into HIV care since the start of the service. Currently, more than 5100 adults are on HAART.

### Study subjects

All selected HIV-infected adults who were taking HAART for at least 1 year at the University of Gondar comprehensive specialized referral hospital ART clinic during the study period were the study subjects.

### Sample size and sampling technique

The sample size was determined using single population proportion formula considering the assumption of 95% level of confidence, 5% of marginal error, and by taking the proportion of immunological and virological failure (30%) reported by a previous study [27]. With these assumptions, the sample size was calculated to be 323. During the 2-month study period, viral load testing was done for 660 HIV-infected adults on HAART. Using systematic random sampling techniques, every other HIV-infected adult on HAART was selected and enrolled in to the study. The first sample order was selected by lottery method from the first two consecutive HIV-infected adults.

### Operational definition

Immunological status of the study participants, as favorable (> 400 cells/mm^3^ for male and > 466 cells/mm^3^ for female) and unfavorable (< 400 cells/mm^3^ for male and < 466 cells/mm^3^ for female), was defined based on the reference interval of CD4^+^ cell count established for the population residing northwest Ethiopia [28]. Virological suppression was considered when the HIV-RNA level is < 1000 copies/μl at any point after 6 months of treatment with HAART [29].

### Data collection tools and procedures

Sociodemographic and socioeconomic data were collected using a structured questionnaire via a face-to-face interview. Clinical data such as baseline WHO clinical stage, CD4 cell count, history of opportunistic infection, TB treatment history, body mass index (BMI), eligibility criteria, and duration since ART initiation were collected by reviewing the medical records of study participants.

### Plasma sample collection and viral load determination

Four milliliters of blood sample was collected in an EDTA tube from each study participant for VL determination. The blood sample was centrifuged at 3000 rpm to obtain plasma, then 1100 μl of plasma was added to the sample tube (S-tube), and it was processed by using COBAS® Ampliprep/COBAS® TaqMan® analyzer (Roach Diagnostics, USA). This test is a nucleic acid amplification test for the quantitation of HIV RNA in human plasma. Nucleic acid isolation from the plasma sample is automated using the COBAS® AmpliPrep Instrument, and then the amplification and automated detection is done using the COBAS® TaqMan® Analyzer. The test is based on three major processes: specimen preparation to isolate HIV-1 RNA, reverse transcription of the target RNA to generate complementary deoxyribonucleic acid (cDNA), and simultaneous polymerase chain reaction amplification of target cDNA and detection and quantification of viral nucleic acid a dual-labeled fluorescent oligonucleotide probe specific to the target.

### Blood sample collection and CD4 cell counting

Three milliliters of blood was collected in EDTA tube for CD4 cell determination. Fifty microliters of whole blood was added to the CD4 reagent tube, and it was incubated, then 50 μl of 5% formaldehyde was added and processed in the Beckman Dickson FACS Calibur^TM^ analyzer (BD Biosciences, San Jose, CA, USA). BD FACS Caliber reagents are provided as complete kits that streamline CD4 cell counting. These kits contain ready-to-use tubes with premeasured antibodies and beads for absolute counting, fixative solution, and software that enables automated analysis without operator intervention.

### Data quality control

The reliability of the study findings was guaranteed by implementing quality control (QC) measures throughout the whole laboratory process. All materials, equipment, and procedures were adequately controlled. The FACS Calibur^TM^ and COBAS® Ampliprep/COBAS® TaqMan® analyzer were checked for reproducibility and performance by using quality control materials. Pre-analytical, analytical, and post-analytical quality assurance measures were strictly followed as per the standard operating procedures (SOPs). A pretest was also done among 16 HIV-infected adults before the actual data collection to check the validity of the questionnaire.

### Data processing and analysis

The data were cleaned, edited, and checked for completeness. They were then entered to the EPI info version 7.0 and transferred to the SPSS version 20 statistical package for analysis. Descriptive summary statistics were done and presented in the tables. A binary logistic regression model was fitted to identify factors associated with the outcome variables. Odds ratio (OR) and its 95% confidence interval (CI) were computed to assess the strength of association and statistical significance. Variables having *p* value less than or equal to 0.2 in the bivariable binary logistic regression analysis were included in the multivariable binary logistic regression analysis to control confounding factors. Variables having a *p* value of less than 0.05 in the multivariable binary logistic regression model were considered to be statistically significant.

## Results

### Sociodemographic characteristics of study participants

All of the selected study participants, 323 HIV-infected adults on HAART, were included in the analysis. The majority of the study participants were urban dwellers (76.5%) and female (61.6%). Regarding age, the current median (interquartile range (IQR)) age of the study participants was 39 years (33–46 years), with the minimum and maximum ages of 18 and 80 years, respectively. The majority, 57.3%, of the study participants were enrolled to the HIV care within the age range of 26–40 years**.**

### Clinical- and treatment-related characteristics

Of the total study participants, 59% were at WHO clinical stage III, and 46.8% of patients took HAART for 6–10 years. At the initiation of ART, 43% were underweighted (< 18.5 kg/m^2^), while only 18% were underweighted during their VL testing. About 26% of the study participants had a past opportunistic infection at enrollment, of which, 10.2% were infected with tuberculosis. For more than half (58.2%) of the study participants, the eligibility of ART initiation was determined by CD4 count. The baseline median (IQR) of CD4 level was 191 (110–291) cells/mm^3^. The majority, (85.8%), of participants were “working” functional status at baseline (Table 1).

**Table 1:** Clinical- and treatment-related characteristics of HIV-infected adults on HAART at the University of Gondar comprehensive specialized referral hospital

Variable	Category	Frequency (%)
Duration since HIV confirmed (year)	1–4	47 (14.5)
	5–10	187 (57.9)
	> 10	89 (27.6)
Past opportunistic infection at enrollment	Yes	84 (26)
	No	239 (74)
Duration from enrollment to eligibility (year)	≥ 1	31 (9.6)
	< 1	292 (90.4)
Baseline WHO stage	Stages I and II	88 (27.3)
	Stage III	191 (59.1)
	Stage IV	44 (13.6)
Duration since ART initiation (year)	1–5	97 (30)
	6–10	151 (46.8)
	> 10	75 (23.2)
Baseline BMI (kg/m^2^)	< 18.5	139 (43)
	≥ 18.5	184 (57)
TB treatment history at enrollment	Yes	33 (10.2)
	No	290 (89.8)
Reasons for eligibility	Clinical	16 (5)
	CD4 count	188 (58.2)
	Total lymphocyte count	1 (0.3)
	Transfer in	2 (0.6)
	Test and treat	7 (2.2)
	Clinical and CD4 count	109 (33.7)
Any medication other than ART	Yes	74 (22.9)
	No	249 (77.1)
Baseline BMI (kg/m^2^)	< 18.5	139 (43)
	≥ 18.5	184 (57)
Baseline anemic status	Anemic	76 (23.5)
	Non-anemic	247 (76.5)
Baseline CD4 count (cell/mm^3^)	< 200	176 (54.5)
	200–499	111 (34.4)
	> 499	36 (11.1)
Baseline functional status	Working	277 (85.8)
	Ambulatory and bedridden	46 (14.2)
BMI at VL testing (kg/m^2^)	< 18.5	58 (18)
	≥ 18.5	265 (82)
CD4 at VL testing (cell/mm^3^)	< 350	106 (32.8)
	350–499	66 (20.4)
	> 499	151 (46.8)

*ART* antiretroviral therapy, *VL* viral load, *WHO* World Health Organization, *BMI* body mass index, *CD4* cluster of differentiation, *Tb* tuberculosis, *HAART* highly active antiretroviral therapy

### Level of immunologic and virological status

Virological suppression was found in 265 of participants, 82% (95% CI 77.7, 86.1). Whereas favorable immunological status (> 400 cells/mm^3^ for male and > 466 cells/mm^3^ for female) was observed in 171, 52.9% (95% CI 47.4, 58.8) of the study participants. The median CD4^+^ cell count was increased from a baseline value of 191 cells/mm^3^ to 470 cells/mm^3^ at the time of VL testing.

### Factors associated with immunological status

In the bivariable binary logistic regression analysis, baseline BMI, baseline WHO stage, baseline CD4 count, residence, and current age were significantly associated with favorable immunological status. Besides, age at enrollment and duration since ART initiation were the candidate to be included in the multivariable binary logistic regression model. However, in the multivariable binary logistic regression analysis controlling the possible cofounders, baseline CD4 count of 200–499 cells/mm^3^ (AOR = 2.7; 95%CI 1.58, 4.62), baseline CD4 count of > 499 cells/mm^3^ (AOR = 9.6; 95% CI 3.06, 30.07), urban residence (AOR = 1.90; 95% CI 1.05, 3.43), and age at enrollment of 26–40 years old (AOR = 0.53; 95% CI 0.15, 0.83) were significantly associated with favorable immunological status (Table 2).

**Table 2:** Factors associated with immunological status of HIV-infected adults on HAART at the University of Gondar specialized referral hospital

Variables		Immunological status		COR (95% CI)	AOR (95% CI)
		Favorable immunological status	Unfavorable immunological status		
Baseline BMI (kg/m^2^)	< 18.5	65 (46.8)	74 (53.2)	1.00	-
	≥ 18.5	106 (57.6)	78 (42.4)	1.55 (0.99, 2.41)	-
Baseline WHO stage	Stages I & II	57 (64.8)	31 (35.2)	2.42 (1.16, 5.07)	-
	Stage III	95 (49.7)	96 (50.3)	1.30(0.67, 2.52)	-
	Stage IV	19 (43.2)	25 (56.8)	1.00	-
Duration since ART initiation (year)	1–5	48 (49.5)	49 (50.5)	1.05 (0.55, 2.00)	-
	6–10	78 (51.7)	73 (48.3)	0.68 (0.54, 1.12)	-
	> 10	45 (60)	30 (40)	1.00	-
Baseline CD4 count (cell/mm^3^)	< 200	69 (39.2)	107 (60.8)	1.00	1.00
	200–499	70 (63.1)	41 (36.9)	2.65 (1.62, 4.32)	2.7 (1.58, 4.62)*
	> 499	32 (88.9)	4 (11.1)	12.61 (4.20, 36.63)	9.59 (3.06, 30.07)*
Patient address	Urban	139 (56.3)	108 (43.7)	1.77 (1.05, 2.98)	1.90 (1.05, 3.43)*
	Rural	32 (42.1)	44 (57.9)	1.00	1.00
Current age (year)	≤ 30	39 (66.1)	20 (33.9)	2.04 (1.04, 4.01)	-
	31–45	87 (50.6)	85 (49.4)	1.07 (0.64, 1.77)	-
	> 45	45 (48.9)	47 (51.1)	1.00	-
Age at enrollment (year)	≤ 25	52 (64.2)	29 (35.8)	1.30 (0.65, 2.61)	0.59 (0.17, 2.10)
	26–40	86 (46.5)	99 (53.5)	0.63 (0.35, 1.15)	0.53 (0.15, 0.83)*
	> 40	33 (57.9)	24 (42.1)	1.00	1.00

*COR* crude odds ratio, *AOR* adjusted odds ratio; *ART* antiretroviral therapy, *CD4* cluster of differentiation 4, *BMI* body mass index, *WHO* World health Organization, *HAART* Highly active antiretroviral therapy

*Significantly associated at *P* value < 0.05

### Factors associated with virological suppression

In multivariable binary logistic regression analysis, CD4 cell count between 350–499 cells/mm^3^ (AOR = 2.56; 95% CI 1.14, 5.75) and > 499 cells/mm^3^ (AOR = 7.71; 95% CI 3.48, 17.09) at VL testing and current age > 45 years old (AOR = 5.99; 95% CI 2.12, 16.91) were significantly associated with virological suppression (Table 3).

**Table 3:** Factors associated with virological suppression of HIV-infected adults on HAART

Variable		Virological status		COR (95% CI)	AOR (95% CI)
		Virological suppression	No virological suppression		
Current age (years)	≤ 30	44	15	1.00	1.00
	31–45	137	35	1.33(0.67, 2.67)	2.06 (0.93, 4.53)
	> 45	84	8	3.58 (1.4, 9.10)	5.99 (2.12, 16.91)*
Age at enrollment (year)	≤ 25	61	20	1.00	-
	26–40	150	35	1.40 (0.75, 2.62)	-
	> 40	54	3	5.90 (1.66, 20.96)	-
Duration since ART initiation (year)	1–5	78	19	1.00	
	6–10	123	28	1.17 (0.55 2.48)	-
	> 10	64	11	1.37 (0.60, 3.10)	-
Baseline CD4 count (cell/mm^3^)	< 200	143	33	1.00	-
	200–499	94	17	1.27 (0.67, 2.42)	-
	> 499	28	8	0.80 (0.34, 1.93)	-
CD4 count at VL testing (cell/mm^3^)	< 350	70	36	1.00	1.00
	350–499	55	11	2.57 (1.20, 5.51)	2.56 (1.14, 5.75)*
	> 499	140	11	6.54 (3.14, 13.63)	7.71 (3.48, 17.09)*
Baseline anemic status	Non-anemic	158	28	1.00	-
	Anemic	107	30	0.63 (0.35, 1.11)	-
Baseline functional status	Working	228	49	1.00	-
	Ambulatory and bedridden	37	9	0.88 (0.40, 1.94)	
Baseline BMI (kg/m^2^)	< 18.5	113	26	1.00	-
	≥ 18.5	152	32	1.09 (0.61, 1.93)	
Baseline WHO stage	Stages I and II	77	11	1.00	-
	Stage III	154	37	0.59 (0.28, 1.23)	-
	Stage IV	34	10	0.48 (0.18, 1.25)	-
Initial regimen	D4T-3TC-based	27	7	1.00	-
	AZT-3TC-based	118	25	1.22 (0.48, 3.12)	-
	TDF-3TC-based	120	26	1.19 (0.47, 3.04)	-

*COR* crude odds ratio, *AOR* adjusted odds ratio, *ART* antiretroviral therapy, *CD4* cluster of differentiation 4, *VL* viral load, *BMI* body mass index, *WHO* World Health Organization, *HAART* highly active antiretroviral therapy

*Significantly associated at *p* value < 0.05

## Discussion

The scale-up of ART service in Ethiopia has been one of the greatest achievements of the HIV program in the last one decade. HAART has been consistently reported to suppress HIV RNA to the level below the limit of detection, and has reduced the risk of clinical progression [30, 31]. Despite these successes, treatment failure due to drug resistance and poor adherence poses a challenge to the ART program. For this reason, the WHO recommends routine VL testing for monitoring ART.

In this study, 82% (95% CI 77.7, 86.1) of the study participants achieved virological suppression, indicating the success of the ART program and suggesting that the country is progressing towards achieving the third 90–90–90 target of the UNAIDS. Despite this achievement, there are still a considerable proportion of patients (18%) with high VL which needs monthly follow-up and repeated VL testing after 3–6 months of enhanced adherence support [10]. These groups of patients are classified as suspected virological failure because they are at higher risk of morbidity and mortality [15, 32]. Therefore, the finding of this study highlighted the importance of improved access to VL monitoring and prompt action to optimize treatment regimen for patients with high VL.

Even though the 82% of virological suppression in this study differs from previous studies, it is consistent with a study from Tanzania (79.1%) [27]. This result is lower than the finding from previous studies in Nepal (90%) [33], Addis Ababa, Ethiopia (90%) [34], Ghana (89.6%) [35], and South Africa (94%) [36]. Similarly, the Ethiopian Public Health Institute reported that 88.1% of patients on first-line treatment showed virological suppression in the country, with a regional variation [37]. In this study, HIV-infected adults enrolled to ART care in 2005, where ART service was started in the hospital, might have participated in the study. These participants could initiate ART medication at the advanced stage of the disease which affects the treatment response [38]. Remarkably, in our study, 57.2 % of study participants were initiated ART at WHO stage 3 or 4 defining conditions, whereas WHO recommends that earlier initiation of ART would improve treatment response and help countries to achieve the 2020 target for virological suppression [39].

Favorable immunologic status was observed among 52.9% (95% CI 47.4, 58.8) of the study participants. This finding is lower than reports from Nepal (62.83%) [33], southern Ethiopia (82.4%) [40], and Debre Markos, Ethiopia (79%) [41]. The variation in the immunologic status of the study participants with other studies might be attributable to the fact that other studies presented for comparison to our finding defined immunological recovery differently. In contrary, our finding is higher than study reports from Oromia, Ethiopia (32%) [42] and Tanzania (43.1%) [27]. Most patients in these two studies initiated treatment lately which can affect the immune reconstitution [43].

In this study, it is noted that there is a discrepancy between the immunological status (52.9%) and the virological suppression (82%). For some of the study participants, the HIV RNA plasma level is below the limit of detection, but the CD4^+^ cell count response is blunted. Some others exhibited a different pattern of discordant response characterized by a sustained CD4^+^ cell response despite persistent viremia. Similarly, studies also reported such type of results [44, 45]. The possible reason for discordant result might be late initiation of ART, HIV-related depletion of T cells, persistent immune activation and exhaustion of T cell due to microbial translocation, long-term impact of HIV on thymus function and its output, and lymph node fibrosis [44, 46].

Current age and CD4 cell count at VL measurement were associated with virological suppression. Accordingly, the odds of virological suppression among patients aged > 45 years old were nearly 6 times more likely compared with those aged < 30 years. This finding confirms the previous study from the rural part of South African describing that greater proportion of older adults (90.1%) had good virological response as compared with younger adults [47]. Probably, older patients might demonstrate better medication adherence which would positively influence to achieve optimal outcome of ART medication [48, 49]. Therefore, adherence assessment with different age categories is critical.

The other factors associated with virological suppression is CD4 cell count at the time of VL testing. The odds of virological suppression among patients who had a CD4 cell count of 350–499 and > 499 cells/mm^3^ were 2.56 and 7.71 more likely to respond compared with patients having CD4 count of < 350 cells/mm^3^, respectively. This is due to the fact that as the current CD4 count raises, the duration on ART increases and the VL is suppressed; HIV/AIDS-related mortality might also decline. Evidence also supports that the effect of CD4 count varied strongly by VL and duration of treatment [50]. Similarly, HIV VL needs to be controlled at a lower level to maintain favorable CD4 response while the patient is on HAART [51].

Favorable immunologic status was seen among those participants with high baseline CD4 cell count, urban residence, and age at enrollment within the range of 26–40 years. The odds of favorable immunologic status among patients who had a baseline CD4 cell count of 200–499 cells/mm^3^ and > 499 cells/mm^3^ were 2.7 and 9.6 times higher compared with patients who had a baseline CD4 cell count of < 200 cells/mm^3^. Other studies have also reported that high baseline CD4 cell count was positively associated with a better immunological response in HIV-infected patients taking HAART [52–55]. This finding enlightened the benefits of earlier initiation of ART. For those patients who had a higher baseline CD4 cell count, it might be easier to reach the normal CD4 level within a short period after initiation of treatment. A study in sub-Saharan Africa has indicated an increment in CD4 cell count by 50–100 cells/mm^3^ 1 year after initiation of HAART [56]. The odds of favorable immunologic status among patients who were residing in urban areas was 1.9 times more likely as compared with patients residing in rural areas. This might be due to lifestyle differences like poor feeding practice, as such in the rural setting most of the time they are not feeding as recommended, and compliance barrier for prescribed ART drugs in the rural area [57].

The age of participants is another factor affecting the immunologic status. Those participants who were in the age range of 26–40 years old at enrollment were 47% less likely to have favorable immunologic status compared with those within the age of > 40 years old. A study aimed at exploring the effect of age on immunologic restoration reported that a younger age favors CD4 cell restoration because of preserved thymus function which was contrary to this study [58]. The discrepancy might be related with adherence problem, which is seen mostly in younger aged group of HIV-infected patients. In support of this argument, one study reported that the rate of poor adherence among young adults was twice as high as older individuals did [59].

Although this study was conducted with due diligence and scientific approaches, there were certain limitations. The level of adherence to ART was not assessed, which might have an effect on the outcome of interest. Furthermore, a single VL measurement was taken to estimate the virological suppression which might underestimate the proportion of patients with virological suppression since VL re-suppression could occur if the test is repeated after 3–6 months of enhanced adherence support.

## Conclusion

This study revealed a sub-optimal level of virological suppression, indicating the need for strong commitment to achieve the third 90% target of the UNAIDS 90–90–90 targets by 2020. On the other hand, the immunological status was found to be low. Age > 45 years old and CD4 cell count ≥ 350 cells/mm^3^ were significantly associated with virological suppression. Age at enrollment between 26 and 40 years was negatively associated with immunological status, whereas urban residence and baseline CD4 cell count ≥ 200 cells/mm^3^ were positively associated. Therefore, early initiation of HAART at higher CD4 cell count level is vital to achieve the goal of HAART. For better treatment outcome, younger age groups and those with low baseline CD4 cell count need to be followed cautiously. This finding also generated evidence for tracking the progress towards achieving the third target of the 90–90–90 UNAIDS plan.

## Data Availability

All relevant data are available within the manuscript. In case of need, the data that support the findings of this study are available from the corresponding author on reasonable request.

## Abbreviations

AIDS: Acquired immunodeficiency syndrome
AOR: Adjusted odds ratio
ART: Antiretroviral therapy
BMI: Body mass index
cDNA: Complementary deoxynucleic acid
CD4: Cluster of differentiation 4
CI: Confidence interval
COR: Crude odds ratio
HAART: Highly active antiretroviral therapy
HIV: Human immunodeficiency virus
IQR: Interquartile range
PLWHA: People living with HIV/AIDS
RNA: Ribonucleic acid
SOP: Standard operating procedure
UNAIDS: United Nations Programme on *HIV/AIDS*
VL: *Viral load*
WHO: World Health Organization
